# Supplementary material for: Development and validation of a mitotic catastrophe-related genes prognostic model for breast cancer
Source: PeerJ. 2024 Sep 20;12:e18075. doi: 10.7717/peerj.18075 (PMC11418815; doi:10.7717/peerj.18075)
Supplement: Supplemental Information 3 [file peerj-12-18075-s003.docx]

**Methods for RT-qPCR Analysis**

**1.qPCR Primers**

Tsingke Biotech (Xi’an, China) designed all primers.

| Prime name | Sequence | Tm(℃) |
| --- | --- | --- |
| PLK1-F | AAAGAGATCCCGGAGGTCCTA | 57.27 |
| PLK1-R | GGCTGCGGTGAATGGATATTTC | 57.85 |
| S100B-F | TGGCCCTCATCGACGTTTTC | 57.67 |
| S100B-R | ATGTTCAAAGAACTCGTGGCA | 56.07 |
| IRS2-F | CGGTGAGTTCTACGGGTACAT | 56.65 |
| IRS2-R | TCAGGGTGTATTCATCCAGCG | 57.91 |
| IFNG-F | TCGGTAACTGACTTGAATGTCCA | 57.11 |
| IFNG-R | TCGCTTCCCTGTTTTAGCTGC | 59.93 |
| βactin-F | CATGTACGTTGCTATCCAGGC | 57.48 |
| βactin-R | CTCCTTAATGTCACGCACGAT | 57.26 |

**2.Methods for RT-qPCR Analysis**

**(1)** **RNA extraction**

Total RNA was extracted from cells or tissues using SPARKeasy Improved Tissue/Cell RNA Kit (AC0202, Sparkjade Biotech Co., Ltd., Shandong, China) following the manufacturer’s instructions. Briefly, cells are transferred to a 1.5 mL microcentrifuge tube and washed once in PBS. Next, the sample is centrifuged at 12,000 rpm for 10 seconds at 4°C. The supernatant is discarded, and then 350 μl (<5×10^6^ cells) of RLTPlus is added to the cells. The tube is vigorously shaken for 20 seconds. The sample is then centrifuged at 12,000 rpm for 3 minutes at 4°C. The supernatant is transferred to a DNA purification column. The sample is centrifuged at 12,000 rpm for 60 seconds at 4°C. The filtered liquid is collected, and an equal volume of ethanol (70%) is added. The mixture is transferred to an RNA adsorption column (RA) and centrifuged at 12,000 rpm for 30 seconds, discarding the filtered liquid. 700 μl of Buffer RW1 is added, incubated at room temperature for 1 minute, centrifuged at 12,000 rpm for 30 seconds, and the filtered liquid is discarded. 500 μl of wash buffer (RW) is added, centrifuged at 12,000 rpm for 30 seconds, the filtered liquid is discarded, and the process is repeated once. Next, the sample is centrifuged again at 12,000 rpm for 2 minutes. 30 μl of RNase-Free H2O is added, incubated at room temperature for 1 minute, centrifuged at 12,000 rpm for 1 minute, and stored at -80°C for later use.

**(2) Preparation of cDNA by Reverse Transcription**

cDNA was synthesized using SPARKscript Ⅱ All-in-one RT SuperMix for qPCR (Sparkjade AG0305)Defrost the template RNA on ice, the RNase-Free H2O at room temperature (15-25°C), and quickly place on ice after defrosting. The gDNA Eraser and 2×SPARKscriptII All-in-one qRT SuperMix are placed on ice for use. Before use, each solution is flicked or mixed upside down and centrifuged briefly to collect the remaining liquid from the wall to the bottom of the tube..Add the following components into an RNase-free tube, gently pipette up and down to mix thoroughly, briefly centrifuge to collect at the bottom of the tube.

| Components | Volume |
| --- | --- |
| TotalRNA/mRNA | 1μg(≤9μL) |
| gDNAEraser | 1μL |
| 2×SPARKscriptII All-in-one qRT SuperMix | 10μL |
| RNase-Free H2O | Up to 20μL |

The reaction program is as follows:

| Temperature | Time |
| --- | --- |
| 50℃ | 15min |
| 85℃ | 5sec |
| 4℃ | forever |

Once the reverse transcription was completed, the cDNAs were kept at 4°C until removed and frozen at -80°C.

**(3) Quantitative-PCR (qPCR)**

We performed qPCR using the Quant Studio 7 Pro (Applied Biosystems). RT-qPCR was performed using 2×SYBR Green qPCR Mix (Sparkjade AH0104).

The PCR reaction system is as follows:

| Components | Volume |
| --- | --- |
| 2×SYBRqPCRMix | 10μL |
| DNA/cDNA | 2μL |
| Forward Primer（10μM） | 0.4μL |
| Reverse Primer（10μM） | 0.4μL |
| Reference Dye II | 0.4μL |
| RNaseFreeH2O | Up to 20μL |

The reaction program is as follows:

| Temperature | Time |  |
| --- | --- | --- |
| 94℃ | 3min |  |
| 94℃ | 20sec | 40 cycles |
| 57~60℃ | 20sec |  |
| 72℃ | 30 sec |  |
| Dissociation Stage | Default program |  |

The internal controls were β-actin. Gene expression levels were quantitatively calculated by the 2^-ΔΔCt^ method.
